# Editorial: Cancer Treatment and Early Detection Targeting HER Receptors

**DOI:** 10.3389/fmolb.2022.940055

**Published:** 2022-06-15

**Authors:** Xiaoqing Cai, Libing Zhang, Shengxi Chen

**Affiliations:** ^1^ School of Pharmaceutical Sciences, Sun Yat-Sen University, Guangzhou, China; ^2^ Tianjin Key Laboratory of Molecular Optoelectronic, Department of Chemistry, Tianjin University, Tianjin, China; ^3^ Biodesign Center for Bioenergetics, Arizona State University, Tempe, AZ, United States

**Keywords:** cancer targeted therapy, EGFR, HER family, HER2, HER3

The human epidermal growth factor receptor (HER) family (including EGFR or HER1, HER2, HER3, and HER4) plays an important role in regulating cell proliferation, differentiation, migration and survival. The overexpression or inappropriate activation of HER1-3 is associated with the initiation, development, migration, and invasive properties of many types of cancers. However, overexpression of HER4 is not significantly related to the survival rate of cancer patients. Numerous efforts have been devoted to study structural features, physiological functions and pathological effects of these HER receptors. Three main types of targeted therapies have been successfully developed in the past two decades, which include small molecule drugs that inhibit the tyrosine kinase activity of HER receptors; monoclonal antibodies (mAbs) that target the extracellular domains of these receptors; and antibody-drug conjugates (ADCs) that combine the target specificity of mAbs with the high cytotoxicity of chemotherapeutics. Despite the successful development of therapeutics that target HER1-3, new and diverse cancer treatments are still required to overcome the current challenges, such as emerging of drug resistance and lack of efficacy to patients with low level of HER expression.

HER1 has been validated as an important oncogenic target in non-small cell lung cancer (NSCLC), glioblastoma and breast cancer. HER2 is also known as well-established target for a variety of cancers including breast cancer and gastric cancer. However, HER1-or HER2-targeted therapy has not been well explored in bladder carcinomas. Based on published data, HER1 was overexpressed in 75% of primary bladder cancer (Carlsson et al., 2015), and HER2 was also found to be positive in about 10% of invasive bladder carcinoma (Laé et al., 2010). The review by Chen et al. *Progress in the Research and Targeted Therapy of ErbB/HER Receptors in Urothelial Bladder Cancer* describes the recent studies and clinical trials on HER1-and HER2-targeted therapy in bladder cancer. Systemic therapies for urothelial bladder cancer, including chemotherapy, immunotherapy, targeted therapy and other treatment methods, are also discussed in this review.

The level of HER2-expression is a critical sign, which allow to predict the eligibility of HER2-targeted therapy for patients. In the review of *HER2 Low, Ultra-low, and Novel Complementary Biomarkers: Expanding the Spectrum of HER2 Positivity in Breast Cancer*, Venetis et al. illustrate the current progress of HER2-targeting therapy in breast cancers with low and ultra-low levels of HER2 expression, which include cancer vaccines, antibody-drug conjugates, and bispecific antibodies.

ADCs as a novel therapeutic paradigm with the best combination of the desirable properties of mAbs and cytotoxic payloads, are rapidly expanding fields of cancer targeted therapy. HER2-directed ADCs have achieved striking clinical success, with trastuzumab-emtansine (T-DM1) and trastuzumab-deruxtecan (DS-8201a) approved by US FDA (Food and Drug Administration) and disitamab vedotin (RC48) approved by China NMPA (National Medical Products Administration). Yu et al. summarized these three approved ADCs and a number of HER1-, HER2-, and HER3-targeted ADCs that are currently under clinical trials in *Antibody-Drug Conjugates Targeting the Human Epidermal Growth Factor Receptor Family in Cancers*. This review also discusses some of the major challenges for ADC development and provides new insights into the possible future directions in this area.

One HER3-targeted ADC (Patritumab deruxtecan) was approved by the FDA for the treatment of locally advanced or metastatic, EGFR-mutant non–small cell lung cancer (NSCLC) patients. To overcome drug resistance to current HER-targeted therapies, numerous new agents are being developed, such as HER-targeting multifunctional nanoparticles (Zhang et al., 2017), HER4-targeting inhibitors (El-Gamal et al., 2021), and targeting epidermal growth factor (EGF)-like proteins. In the research article of *Pan-Cancer Analysis Identified CD93 as a Valuable Biomarker for Predicting Patient Prognosis and Immunotherapy Response*, Tong et al. used bioinformatics approaches to discover the correlation of the expression of CD93 with the tumor stage and immune infiltration. CD93 is a transmembrane protein containing an epidermal growth factor (EGF)-like domain (Kao et al., 2012).

Taken together, this special Research Topic gives an overview on the current development of cancer therapeutics targeting HER receptors. It also highlights the interesting insights into new and complementary approaches for overcoming the challenges of HER-targeted therapies.
